# Asiatic Black Bear–Human Conflict: A Case Study from Guthichaur Rural Municipality, Jumla, Nepal

**DOI:** 10.3390/ani14081206

**Published:** 2024-04-17

**Authors:** Akshay Kumar Rawal, Sachin Timilsina, Subash Gautam, Saurav Lamichhane, Hari Adhikari

**Affiliations:** 1 Institute of Forestry, Pokhara Campus, Tribhuvan University, Pokhara 33700, Nepal; 2 Department of Food and Resource Economics, Faculty of Science, University of Copenhagen, Rolighedsvej 23, 1958 Frederiksberg C, 1165 Copenhagen, Denmark; 3 Seneca Polytechnic, School of Environmental and Civil Engineering Technology, 1750 Finch Avenue East, Toronto, ON M2J 2X5, Canada; 4 Faculty of Forestry, Agriculture and Forestry University, Hetauda 44100, Nepal; 5 Department of Geosciences and Geography, University of Helsinki, P.O. Box 64, FI-00014 Helsinki, Finland; 6 Forest Nepal, Amar Marg 88, C3534, Butwal 32907, Nepal

**Keywords:** human–black bear conflict, compensation, depredation, habitat, Himalayan black bear

## Abstract

**Simple Summary:**

This research paper presents a comprehensive study of the patterns of conflicts between humans and Asiatic black bears (Ursus thibetanus) in the Guthichaur rural municipality, Jumla, Nepal. Through semi-structured interviews with villagers, focus group discussions, and key informant interviews, this study explores the extent of crop damage, livestock depredation, and human injuries caused by black bears from 2009 to 2019. It was found that crop damage was the most significant form of conflict, followed by livestock depredation and human casualties. This study identifies anthropogenic activities, such as human encroachment into bear habitats and agricultural practices near forests, as primary drivers of these conflicts. Importantly, this research proposes measures to mitigate these conflicts, including initiating compensation schemes for losses, establishing electric fences for crop protection, and launching educational programs. These recommendations, rooted in local practices and conservation efforts, show promise for managing conflicts in regions facing similar challenges with black bears. This paper fills a critical gap in understanding the dynamics of human–bear conflicts in Nepal, contributing valuable insights into wildlife management and conservation strategies. Its findings are significant for researchers, policymakers, and conservationists aiming to develop sustainable solutions for human–wildlife coexistence.

**Abstract:**

Our study assessed patterns of Asiatic black bear (*Ursus thibetanus*)–human conflicts within the Guthichaur rural municipality, Jumla, Nepal. Through semi-structured interviews with villagers, focus group discussions (FGDs), and key informant interviews (KIIs), we gathered black bear–human conflict information from 2009 to 2019. We identified three primary types of black bear–human interactions: crop damage, livestock depredation, and human injuries. Of these, crop damage (77.03%) emerged as the most prevalent issue. Notably, peak occurrences were observed during autumn (September–October) typically between 9 PM and 3 AM. Livestock depredations were more frequent during nighttime in April–August, with cows/ox (70.12%) being the most depredated animal. Our data also revealed five recorded cases of black bear attacks on humans, which transpired from September to October, primarily in farmland areas in varying years. Despite a prevailing negative perception of bears, a notable level of support exists for their conservation efforts among local communities. Furthermore, these conflicts could be mitigated by reinforcing indigenous crop protection methods and implementing targeted mitigation strategies, as observed in other regions with successful black bear–human interaction management.

## 1. Introduction

Human–wildlife conflicts (HWCs) are widespread globally; however, they are particularly prevalent and frequent in less developed regions, specifically in African and Asian countries [1,2], where a substantial portion of the human population depends on agriculture and livestock [1,3]. It arises when the mutual interactions between humans and wildlife harm both parties, stemming from the competition for space and resources [4,5]. The loss of human life and damage to property and crops due to HWC can cost the affected communities considerably [3]. The Asiatic black bear (Ursus thibetanus) is widely distributed in South and East Asia [6], regularly interacts with rural people [6], and is frequently involved in conflicts with humans [7,8]. Bears can cause crop damage [9,10], livestock depredation [7,11], beehive loss [12], and even human injuries or deaths [13,14]. The Asiatic black bear, native to 20 Asian countries, including Nepal, is listed as vulnerable on the International Union for Conservation of Nature Red List and in Appendix A of the Convention on International Trade in Endangered Species of Wild Fauna and Flora. The Asiatic black bear is also recognized as nationally endangered in Nepal [15]. Out of eight species of bear across the world, three species are found in Nepal: the Asiatic black bear (*Ursus thibetanus)*, the brown bear (*Ursus arctos*), and the sloth bear (*Melursus ursinus*) [16].

Asiatic black bears in Nepal are scattered broadly across mid- to high-elevation mountains (1400–4000 m), including 13 protected areas, and have an estimated small population size of about 500 individuals, which is observed to be declining [17]. This species’ available suitable habitat space is estimated to have shrunk by approximately 30% over the past ten years [18]. Furthermore, habitat fragmentation, habitat loss and destruction, habitat encroachment for agricultural land, illegal hunting, logging, and human–bear conflict are significant threats to Asiatic black bears across their global range [19].

The illegal trade of bear body parts, such as skin and claws, along with the sale of live cubs for bearbaiting, dancing bears, and bile extraction farms, significantly exacerbates human–bear conflicts, threatening the survival of black bears and highlighting critical conservation challenges. In addition, the expansion of the human population increasingly overlaps with established wildlife territories, squeezing wildlife and forcing them to compete for limited space and resources. Because of habitat fragmentation, the animal population is compressed into insular refugees, where animals may be at higher risk of encountering humans and experiencing conflicts over resources to meet their nutritional, ecological, and behavioral requirements [20]. Compared to other wild animals in Nepal, the Asiatic black bear wreaks havoc primarily through livestock depredation, crop (chiefly maize) damage, and human casualties [15]. This is the principal cause of the human–bear conflict in Nepal; in most cases, local people respond with the retaliatory killing of bears [13]. The Asiatic black bear can act aggressively toward humans, especially when it feels threatened. However, it usually prefers to retreat rather than confront humans directly [21]. In a period of four years (2013/14 to 2016/17), black bear issues were reported in 18 wards (previous Village Development Committees) of 11 districts of Nepal; 25 people were wounded, and 3 individuals were killed in those areas [15]. Between 2010 and 2014, bears were responsible for 12% of all wildlife conflicts that resulted in death or injury [22]. Asiatic black bears are globally recognized as vulnerable, but compared to other charismatic and critically endangered species in Nepal, black bears have received little priority from a conservation point of view [17]. On the other hand, Asiatic black bears have become a source of problems for people and face multiple threats; this has been worsened by a lack of information about their status, distribution, and requirements for survival [23], as well as illegal hunting (the bear’s gallbladder traditionally has medicinal value) and retaliatory killing for crop and livestock damages, especially in remote areas like Jumla, where this study was conducted.

Despite existing research, significant gaps remain in understanding human–bear conflict dynamics, especially regarding Asiatic black bear habitat use, behavior, and population in Nepal [19]. With limited studies on Asiatic black bears in Nepal, and no specific investigations undertaken in the Jumla region, this study aims to contribute to the literature by examining the types of conflicts occurring between humans and black bears and exploring potential solutions in Nepal and similar landscapes. Therefore, this comprehensive study of Asiatic black bears in Guthichaur rural municipality, Jumla explores ground-based management interventions to minimize conflicts, maximize options for human–bear coexistence, and contribute to the future existence of this charismatic species within the mountain ecosystem.

## 2. Materials and Methods

### 2.1. Study Area

**Figure 1:**
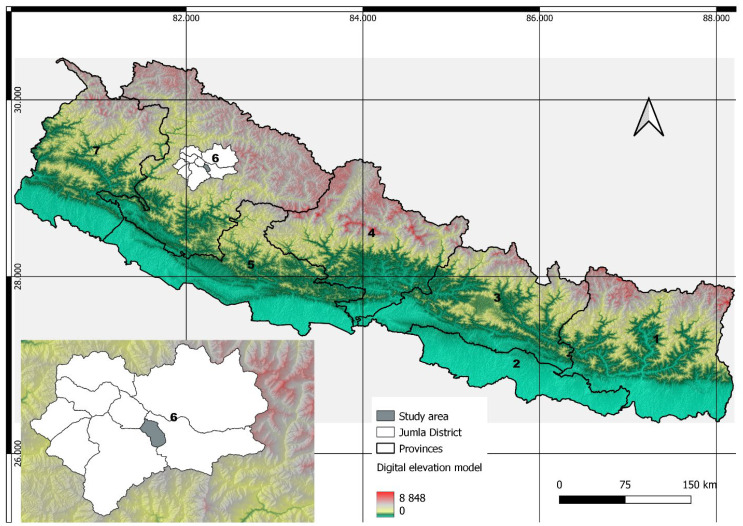
Map of the study area showing seven provinces (1 = Koshi province, 2 = Madhesh province, 3 = Bagmati province, 4 = Gandaki province, 5 = Lumbini province, 6 = Karnali province, and 7 = Sudurpashchim province) of Nepal and the Guthichaur rural municipality.

This study was conducted in the Guthichaur rural municipality of the Jumla district of Province 6 of Nepal (Figure 1). The Jumla district includes the Guthichaur rural municipality to the east, the Chandannath municipality and Tatopani rural municipality to the west, the Patarasi rural municipality to the north, and the Jajarkot district to the south. The total area of the Guthichaur rural municipality is 427 square km (164.86 sq mi). This rural municipality is divided into five wards. This study was centered in Ward 5, which includes four villages: Dhita, Depalgaun, Phoie, and Kumdi. This region lies in the southeast part of the Jumla district at approximately 29°7′ N to 29°16′ N latitude and 82°12′ E to 82°14′ E longitude. Jumla is a Himalayan mountainous region with elevations ranging from 915 to 4679 m. Jumla is abundant in biological diversity and has numerous vegetation types due to its geography and climatic variation. The tree species in the region include pinus species, dhupi (*Juniper incana*), gurans (*Rhododendron arboretum*), bhojpatra (*Betula utilis*), okhar (*Juglans regia*), and khasru (*Quercus semicarpifolia*). The fauna includes the Asiatic black bear (*Ursus thibetanus*), Himalayan goral (*Naemorhedus goral*), leopard (*Panthera pandus*), wild boar (*Sus scrofa*), and wolf (*Canis lupus*). The bird species include the danphe (*Lophophorus impejanus*), kalij (*Lophura leucomelanos*), partridge (*Perdix perdix*), dove (*Columba species*), and pigeon (*Columba livia domestica*).

### 2.2. Research Sampling Design

We contacted the Divisional Forest Office (DFO) in Jumla before the fieldwork, as they are the main authority responsible for addressing wildlife damage outside protected areas and providing relief to victims [24]. The meeting with the DFO to identify the study site was based on conflict cases with the Asiatic black bear. The meeting and official records suggested selecting the Guthichaur rural municipality, Ward 5, for this research based on the higher level of damage caused by the black bear there. After selecting the municipality, we approached the ward office, the smallest political unit of the local government, to collect the household information. Out of 498 households in the ward, 84 were chosen for interviews using a stratified random sampling method. We stratified the sample based on village location, with the number of households selected from each village proportional to its population size. This method ensured representation from all affected areas in the ward. To ensure randomness and representation within settlements, we employed Slovin’s [25] formula (Equation (1)).

(1) $$n=\frac{N}{\left(1+{Ne}^{2}\right)}$$

where *n* is the number of samples, *N* is the total population, and *e* is the margin of error.

We conducted eight focus group discussions (FGDs), five key informant interviews (KIIs), and 84 questionnaire surveys to collect primary information about the damage caused by the black bear over ten years. Incorporating feedback from the DFO staff, FGD, and KII, questionnaires were prepared in Nepali based on anecdotal information concerning the damages (crop, livestock, and humans) caused by the Asiatic black bear. The KIIs were purposively chosen based on their expertise, involvement in conflict resolution, and direct experience with wildlife damages. Focus group participants were selected using purposive and random sampling methods, ensuring a diverse representation of community members. We also confirm no overlap between participants in the questionnaire survey, KIIs, and FGDs. A local field assistant, knowledgeable about the study site, was hired part-time to aid in data collection. Trained to conduct face-to-face interviews, the assistant accompanied the team throughout the fieldwork. Secondary information was obtained from various sources, such as documents from the DFO office, the ward office at a local level, and related literature.

### 2.3. Data Collection

#### 2.3.1. Key Informant Interviews (KIIs)

Key informants who can provide the investigator with insight into corroboratory evidence are often critical to research success [26]. Five individuals were interviewed: a rural municipality staff member, a person attacked by a black bear, a local herder, a teacher, and a ward staff member. Each interviewee was presented with a separate checklist relevant to their experience (1–2 h per interview). A semi-structured yet informal interview method featuring open-ended questions was employed to gather participants’ perspectives on black bear damage and conservation practices. For example, the victim attacked by the bear was asked about their willingness to accept and engage in the relief or compensation process and any adopted strategies (e.g., crop guarding and electric fencing). Livestock owners were asked about the trend of damages by bears in the jungle, and ward representatives were asked about conflict issues, including human casualties, program planning, and budget allocation to reduce wildlife damages, including support to victims at the local level. The KII helped in identifying the most affected settlements, understanding the dynamics of village conservation programs, and learning about their collective efforts. We ascertained the livestock categories, identified seasonal pastures, and mapped the location of livestock depredation with the help of KIIs.

#### 2.3.2. Focus Group Discussions (FGDs)

Altogether, eight FGD meetings were organized in the most bear-affected areas, and discussions took place with local communities. The participants included a group of women frequently involved in agricultural and forestry activities and herders in each village in the study area. Each group comprised five to seven members, and the time spent on the discussion was 1–1.5 h. The information was collected in a participatory manner. We facilitated the discussion process with a checklist of issues, using standard participatory rural appraisal tools like resource maps and trend lines/timelines to understand the local people’s livelihood activities, the status of the black bear in recent years, and the locals’ conservation attitudes. This also allowed us to check the reliability of data collected from each household. The meeting provided equal opportunities for interaction among respondents of similar backgrounds, and the discussion provided information used to verify or cross-check the information obtained from the DFO records. Also, the information obtained from the group discussion was used to improve the household survey questionnaires and to further interpret the results.

#### 2.3.3. Household Surveys

Using feedback from FGDs and KIIs, we developed questionnaires concerning damage (crop, livestock, and humans) caused by Asiatic black bears. Door-to-door visits were made to conduct face-to-face interviews with available members of households [26]. A total of 84 interviews were conducted after obtaining verbal consent to participate. The household survey demographics are presented in Table 1. Even though most of the respondents expressed dissatisfaction with the existing relief/compensation schemes for wildlife damages, none refused the interview. All interviewees were assured that their responses would be kept confidential and used only for research purposes. No identifiable information will be disclosed in any reports or publications. Each respondent’s consent (as “yes”) was documented on the questionnaire after the assurance of confidentiality and privacy of information was delivered [27]. Some interviews were only possible after two or more visits to meet concerned people due to their busy livelihood schedules. The interview was conducted in an informal setting, mainly in the interviewee’s courtyard, often in the presence of other family members and neighbors. Each interview took 2–2.5 h. Questions that were not understood were explained to the interviewees. The time taken varied depending on the level of comprehension of the interviewees. The questionnaire covered a range of topics, including the respondent’s demographics (name, gender, age, education, occupation), agricultural details (crop types, livestock holdings and damages), human–bear conflict experiences (human injuries, if any), and their knowledge of preventative measures and potential relief/compensation schemes. The semi-structured questionnaire gathered information about local communities’ attitudes toward black bear conservation. The final questionnaire consisted of five main sections: (i) the demographic characteristics of the respondent; (ii) crop status, including a calendar of major crops, the amount of agricultural production and loss, and the behavior of the black bear related to crop damage; (iii) livestock status, including details of livestock holding, grazing, and livestock depredation; (iv) details of human casualties; and (v) the respondent’s attitude toward black bear conservation. In assessing conservation attitudes, we drew upon the influence of Theory of Planned Behaviors [28] attitudes on behaviors with subjective norms, perceived control, and intention. Within this framework, attitudes are understood to comprise cognitive (beliefs and perceptions about conservation), affective (emotional responses toward conservation issues), and behavioral components (intentions and actions related to conservation), as outlined by [29].

#### 2.3.4. Field-Based Evidence and Secondary Sources

Initial details about human–bear conflicts, including dates, locations, and types of incidents, were obtained directly from conflict/wildlife attack records maintained by the DFO and local news records, specifically from Karnali F.M. radio. A comprehensive review of existing data was conducted to complement the primary data collection. This included published reports, newsletters, journal articles, books, master’s and doctoral theses, and annual reports containing survey data and documentaries.

### 2.4. Data Analysis

Several open- and close-ended questions (Appendix A.1, Appendix A.2, Appendix A.3, Appendix A.4 and Appendix A.5) were analyzed using IBM Statistical Package for the Social Sciences (SPSS) Statistics 28.0.1. For attitude assessment, the responses of the respondents were measured in 3 different levels, namely “strongly agree”, “Moderately agree” (or okay), and “disagree”, in a Likert format. In addition, the likelihood ratio chi-square test was used to test the significance of variables with statements of local people’s attitudes toward black-bear conservation and contributions to environmental quality.

## 3. Results

### 3.1. Crop Damage

A proportion of 85% of the respondents (71 households out of 84) reported crop damage between 2009 and 2019. Out of the 71 households that suffered from crop damage, 97% (69 homes) reported suffering crop damage by black bears. Besides the black bear, however, porcupines, jackals, wild boars, gorals, monkeys, snakes, and mice caused damage. Out of the total crops affected across all surveyed households (37,386 kg, or 24.56% of total production) (Table 2), 77.03% were damaged by black bears, followed by porcupines (19.54%), jackals (1.99%), wild boars (0.58%), gorals (0.09%), monkeys (0.13%), and mice (0.64%).

The twenty-four-hour day was classified into four different periods—early night (6 p.m.–9 p.m.), midnight (9 p.m.–3 a.m.), early morning (3 a.m.–6 a.m.), and day (6 a.m.–6 p.m.)—to examine the bear’s most active period of crop raiding. Out of 69 households that reported crop damage by a black bear, a black bear was stated to be active during the early night (12%), midnight (66%), early morning (16%), and day (6%). Being a nocturnal animal, the black bear’s crop-raiding activities were primarily confined to nighttime (6 p.m.–6 a.m.) (94%).

### 3.2. Livestock Holding and Depredation

A total of 84 households owned 528 livestock, including cows/ox, buffalo, horses, sheep, and goats. The most reared livestock was cows/ox, comprising 61.17% (323) of the total livestock, followed by goats, buffalo, horses, and sheep, at 16.48% (87), 9.28% (49), 7.96% (42), and 5.11% (27), respectively.

Among the four villages in the study area, the maximum number of livestock, 283, was reported by interview participants in Depalgaun village (41.86%), while the minimum number was reported for Dhita village (9.47%). The average number of livestock holdings per village was 105.6 ± 120.34 per household. Based on the sample survey, livestock holdings were reported as 50 in Dhita, 221 in Depalgaun, 129 in Foi, and 128 in Kumdi.

**Figure 2:**
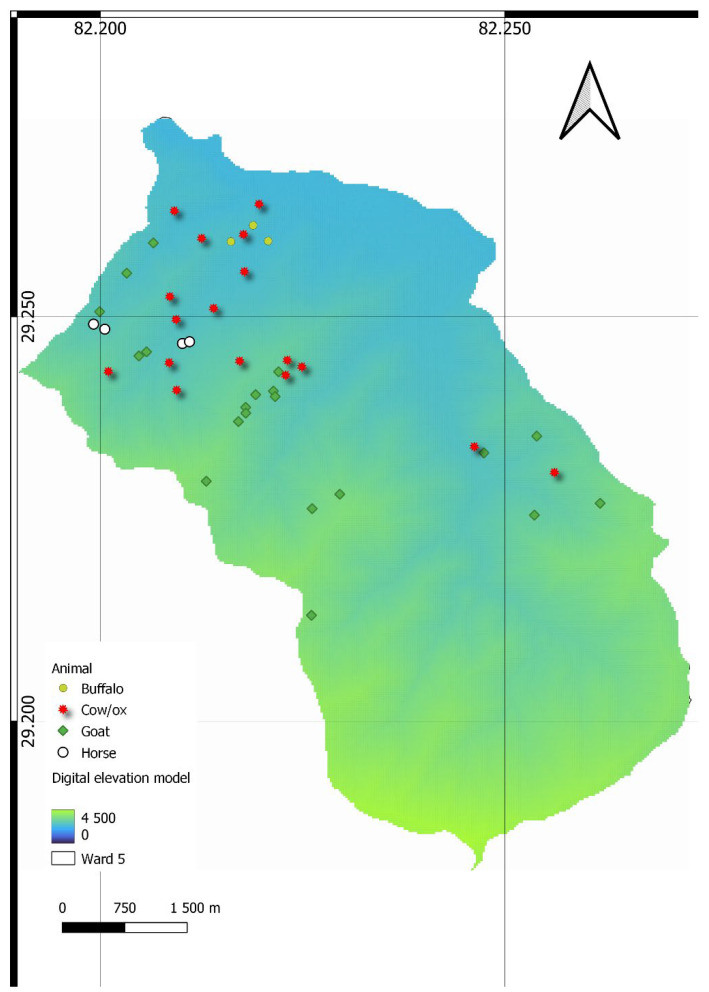
Site of wildlife attacks on livestock.

Out of 84 households surveyed, 55% of the respondents confirmed that they had suffered from livestock depredation by a black bear in the last ten years, while the remaining 45% responded that they had not (Figure 2). The black bear was the only type of wildlife responsible for livestock depredation. The most depredated livestock was cows/ox, constituting 70.12% of the total livestock depredation, followed by goats, buffalo, and horses, at 23.78%, 3.05%, and 3.05%, respectively. The livestock depredation level was found to be 3.57 livestock per affected household (Table 3). Foi village had the highest depredation level (4.92 livestock per household) and was near the forest. In contrast, Depalgaun village had the lowest depredation level (2.18 per household).

**Figure 3:**
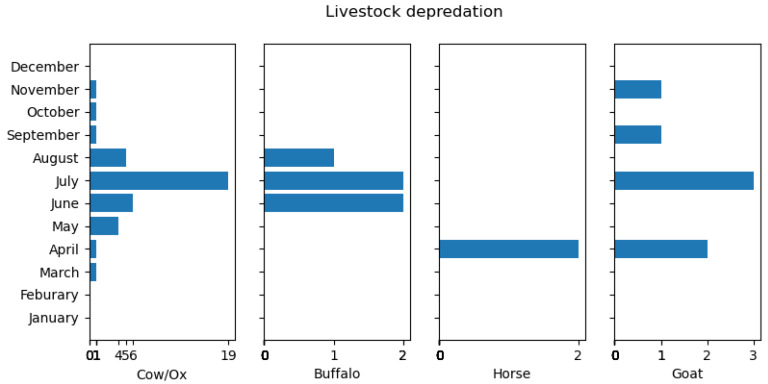
Monthly categorization of livestock depredation (2009–2019).

Livestock depredation in the last ten years occurred most often in July (24); it did not occur in December, January, or February, as shown in Figure 3.

Ninety-four percent of the total attacks on livestock were found in forests and rangeland. Only 4% and 2% of overall attacks on livestock occurred in sheds and cropland, respectively. Most livestock depredation (47%) occurred during nighttime, followed by some attacks during daytime (32%). A proportion of 21% of events were uncertain regarding the time of death, whether during the day or night.

### 3.3. Human Injuries by a Black Bear

In the past decade (2009–2019), records from the rural municipality office indicate three instances of black bear attacks on humans. When interviewed, 14 respondents reported encountering black bears without being attacked. During the field survey, five cases of attack were recorded, including the three previously mentioned cases (Table 4). All attacks occurred in Kumdi village, with four stemming from the same bear, and three in one day, all during September and October. Notably, attacks mostly transpired when victims were in groups.

### 3.4. People’s Attitudes toward Black Bear Conservation

**Figure 4:**
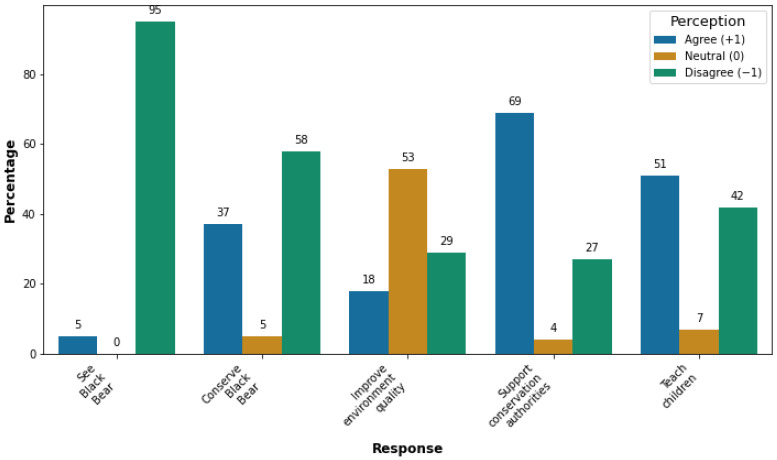
The attitude of respondents toward black bear conservation. Questions asked with the respondent were as follows: (1) Do you think the black bear should be conserved/ protected or have the right to live in a forest? (2) Do you think the black bear improves the environment’s balance/stability/quality? (3) Do you always want to see the black bear in your surroundings/range lands? Do you feel good seeing black bears? (4) Would you support conservation authorities with time or money if they started to conserve black bears in your area? (5) Should we teach our children to help protect black bears?

The survey provided insights into the conservation attitudes of residents. Results indicated a nuanced perspective: (1) 5% of the respondents agreed with the statement that they always want to see black bears in their surroundings/rangelands, and 95% disagreed; (2) 58% of the respondents disagreed with the statement that black bears should be conserved/protected or have the right to live in forests, 5% remained neutral, and 37% agreed; (3) 29% of total respondents disagreed with the statement that the black bear improves the balance/stability/quality of the environment, 53% remained neutral, and 18% agreed; (4) 27% of total respondents disagreed with the statement that they should support conservation authorities in terms of time and money if they start to conserve black bears, 4% remained neutral, 69% agreed, and 27% disagreed; and (5) 42% of total respondents disagreed with the statement that they should teach their children to help in the protection of black bears, 7% remained neutral, and 51% agreed, as shown in Figure 4. Figure 4 shows that local people did not want black bears in their surroundings but supported protection. The results of a chi-square test reveal the following:

(1) There is no significant difference in the perceptions of males and females regarding the conservation of the black bear (χ² = 5.248, df = 2, *p* = 0.073). Specifically, both males and females showed similar levels of support for conserving the bear. However, significant disparities were found between males and females in their perceptions of the role of black bears in improving environmental quality (χ² = 8.386, df = 2, *p* = 0.015). It shows that males tended to exhibit more positive attitudes toward the role of black bears in environmental quality improvement compared to females.
(2) Perception toward conserving black bears did not vary significantly based on the education level of the respondents (λ = 11.230, d.f = 6, *p* = 0.082), suggesting potential differences in perspectives on black bear conservation efforts among individuals with varying levels of education.

## 4. Discussion

### 4.1. Crop Damage

Crop damage by black bears was the main problem (Table 2), similar to the findings of [13] in India, [14] in Pakistan, and [7] in Bhutan. Besides black bears, other wildlife species causing crop damage encompass porcupines, jackals, wild boars, gorals, monkeys, snakes, and mice, mirroring findings from a study conducted by [30] in the Gaurishankar Conservation Area, Nepal. They found maize to be a significant crop damaged by black bears, monkeys, porcupines, gorals, barking deer, and jackals. In addition, maize emerged as the primary crop raided by black bears (Table 2), which is consistent with the results of [11] and [31] in Pakistan, [32] in India, [12] in China, and [7] in Bhutan. This is also evidenced by the frequent sightings of bears in the northern regions of the Dhorpatan Hunting Reserve (DHR), Nepal, drawn by the abundance of maize crops and dense vegetation [33]. This could be because food becomes scarce in forests during autumn, prompting bears to descend to agricultural lands and raid fruit plants and maize crops [11]. Our study shows that black bears were more active during midnight (9 PM–3 AM) and least active in the daytime (6 AM–6 PM), which aligns with the study of [33] in DHR, Nepal. On the contrary, several studies [34,35,36,37,38,39] have indicated that black bear movement patterns, with most of the activity occurring during daylight hours, peak notably in the morning and evening and are lowest at night [35,36,37,38].

Effective crop protection measures are imperative to mitigate potential damage caused by wildlife, including black bears. To safeguard crops from bear intrusion and minimize conflicts between bears and agricultural activities, a variety of protective measures can be implemented. These may include crop guarding techniques and the installation of electric fencing, such as solar-powered electric fences [10]. However, it is essential to evaluate the effectiveness of these measures based on evidence from the literature. Further research is warranted to assess the efficacy of different crop protection strategies and to develop comprehensive approaches for mitigating human–bear conflicts in agricultural settings [10]. Furthermore, research on Asiatic black bear food preferences, feeding habits, and habitat utilization could also help identify sources of conflict and facilitate bear conservation and management [7].

### 4.2. Livestock Holding and Depredation

Cows/ox were the chief livestock depredated by a black bear (Figure 2), which aligns with the study of [39], which states that black bears are the main predators of cattle in the Nanda devi biosphere reserve in India. On the contrary, the study of [33] in DHR found sheep to be primarily depredated livestock, followed by cows and ox. Ref. [14] also shows sheep/goats as the most depredated livestock by black bears in Kaghan Valley, Pakistan. The reason for sheep/goat depredation could be the growing practice of unsupervised livestock rearing, particularly on higher slopes [40]. Similar findings are reported in China [41] and Himachal Pradesh, India [42].

Livestock depredation mainly occurred during July (summer) and was not seen in December, January, and February (winter) (Figure 3), which aligns with the findings of [11,14] in Pakistan. This might be due to the seasonal migration of villagers to temporary residences near forested areas during summer months for crop cultivation and livestock grazing [14]. Also, the other reason might be due to the hibernation or sleep period of the black bear in winter [11], compounded by the practice of stall feeding for livestock due to the limited availability of grass or fodder on the ground [11,40,41,42]. A study performed by [43] in and around Khangchendzonga National Park, Sikkim, reveals that the highest number of conflicts occurred during autumn season. Depredation is highest from April to August in our study (Figure 3) as livestock such as cows, ox, and buffalo are taken to the forest and released for grazing [14]. Moreover, livestock depredation occurred mainly in dense forests and rarely inside the sheds/villages in our study, consistent with the study of [14] in Kaghan Valley, Pakistan [14]. This could be because shepherds are preoccupied with fodder collection or are lax in guarding their herds while grazing in the forest, leading to increased vulnerability to black bear attacks [11]. Livestock depredation could also be attributed to differing grazing practices, such as releasing horses on high-altitude rangelands and allowing goats and sheep to graze with minimal supervision or efforts to locate them by villagers, thereby increasing their vulnerability to predation [7,13]. A study conducted by [13] in Dachigam National Park, Kashmir, India, yielded contrasting results to our own findings. They observed that 3 out of 28 livestock were killed in forests during nighttime, while 19 were killed in cattle sheds or shelters during the same period (2007–2009). Notably, they found a higher incidence of livestock depredation in the winter, contrary to our findings. Potential explanations could include variations in prey availability between the two study areas, differences in predator species composition, or specific management practices employed in Dachigam National Park.

### 4.3. Human Injuries by a Black Bear

Human attacks mainly occurred in September and October (autumn) (Table 4) as black bears moved down into the lowlands, drawn by the smell of ripening fruits such as apples and peaches. A study in Sikkim indicates that bears travel long distances in search of food before hibernation, heightening conflicts with humans due to increased food needs [11]. Human interference in bear habitats and crop cultivation and harvesting near forests were identified as the main factors contributing to these interactions (Table 4), which were found to be consistent with the findings of [14]. In autumn (September–October), bear encounters occurred in farmland where bears foraged on maize crops and in forests where villagers collected non-timber forest products like mushrooms or grazed livestock [12,14]. Some attacks were observed on trails (Table 4), possibly due to increased human movement in autumn for fodder and firewood collection [13]. Black bears mostly attack humans in groups rather than single people alone (Table 4). A study by [26] in CNP also shows that most wildlife attacks, including rhinoceros (*Rhinoceros unicornis*), tigers (*Panthera tigris*), sloth bears (*Melursus ursinus*), elephants (*Elephas maximus*), and wild boars (*Sus scrofa*), occurred when the victims were with a friend or in a group.

### 4.4. People’s Attitudes toward Black Bear Conservation

Our findings suggest that negative attitudes among local communities, shaped by past incidents of livestock attacks, crop raids, and human casualties (Table 2, Table 3 and Table 4 and Figure 3), persist despite their overall support for black bear conservation (Figure 4) [14]. Still, the local people have demanded immediate control of the bears, killing them if necessary. This dichotomy highlights villagers’ reluctance to coexist with black bears in their immediate vicinity. A study by [44] in western Uganda also reported the negative attitude of people toward wildlife when damage by wildlife exceeded the tolerance level. Mitigating such conflicts could improve people’s attitudes toward Asiatic black bears, which, in turn, could result in more effective conservation outcomes for the species [12]. However, the municipality’s economic status has not been strong enough to compensate people for their crops, livestock, and loss of lives. Therefore, to achieve this, education programs [12] and bear conservation strategies [7] should be initiated by officials from national parks (Figure 4). Moreover, recommendations include launching compensation schemes and establishing electric fences for crop protection [10]. Moreover, further research should investigate people’s attitudes toward various forms of mitigation, including compensation for losses to black bears. We also recommend awareness sessions about wildlife’s importance in fostering harmonious coexistence between humans and bears in this ecosystem. Hence, we advocate for collaborative efforts with communities to identify and implement socially acceptable and conservation-friendly measures to mitigate human–bear conflicts.

## 5. Conclusions

This study documents the presence of Asiatic black bears and highlights conflicts with local communities in the Guthichaur rural municipality, Jumla, Nepal. The economic losses from livestock depredation and crop damage were evident. Various factors contributed to livestock depredation, crop damage, and human casualties, warranting urgent interventions, such as initiating insurance schemes for affected communities.

## Figures and Tables

**Table 1:** Demographic characteristics of respondents.

Demographic Characteristics	Number (%)
Respondents	
Male	49 (58.33%)
Female	35 (41.67%)
Age in years	
Young (18–35)	34 (40.47%)
Adult (36–49)	23 (27.38%)
Elderly (50 and above)	27 (32.15%)
Ethnicity	
Brahmin	35 (41.67%))
Chhetri	35 (41.67%)
Thakuri	4 (4.76%)
Dalit	10 (11.9%)
Education	
Illiterate	33 (39.29%)
Basic	29 (34.52%)
Secondary	19 (22.62%)
University	3 (3.57%)
Income source	
Agriculture	18 (21.43%)
Livestock farming	1 (1.19%)
Agriculture and livestock farming	36 (42.86%)
Service	20 (23.81%)
Labor	7 (8.33%)
Local business	2 (2.38%)

**Table 2:** Crop and fruit production and damage.

S. N.	Crop Type	Total Production (kg)	Comprehensive Damage (kg)	Average Damage per Household (kg) ± SD	Black Bear Damage (kg)	Average Black Bear Damage (kg) ± SD
1	Maize	17,116	11,714	139.45 ± 145.68	10,562	125.74 ± 133.24
2	Wheat	14,728	7007	83.42 ± 102.59	6792	80.86 ± 88.12
3	Barley	8605	820	9.76 ± 36.80	820	9.76 ± 19.32
4	Potato	21,260	6065	72.20 ± 85.89	-	-
5	Bean	5994.5	587	6.99 ± 21.39	-	-
6	Rice	36,665	630	7.5 ± 19.39	400	4.76 ± 14.24
7	Buckwheat	1162	343	4.08 ± 13.34	203	2.42 ± 11.23
8	Millet	1180		-	-	-
9	Fruits (apple, peach)	45,500	10,220	121.67 ± 134.76	10,020	119.29 ± 135.34
Total		152,210.5	37,386	445.07	28,797	342.82

The “Total Production” column indicates the total weight of each crop type harvested in kilograms (kg). The “Comprehensive damage” column represents the total weight of crops damaged due to various factors, expressed in kg. Additionally, the “Average damage per household” column shows the average damage per household, calculated by dividing the comprehensive damage by the total number of households surveyed (84). Furthermore, the “Black bear damage” column specifies the portion of damage attributed to black bears, expressed in kg. The “Average Black bear damage” column illustrates the average damage caused by black bears per household, calculated by dividing the black bear damage by the total number of households surveyed (84).

**Table 3:** Total livestock lost during the previous 10-year period (2009–2019).

Village	Number of Households	Cow/Ox	Buffalo	Horse	Sheep	Goat	Total	Average ± SD
Dhita	2	3	0	0	0	4	7	3.5 ± 1.5
Depalgaun	17	24	0	0	0	13	37	2.18 ± 7.61
Foi	12	52	1	2	0	4	59	4.92 ± 17.32
Kumdi	15	36	4	3	0	18	61	4.07 ± 15.45
Total	46	115	5	5	0	39	164	3.57 ± 13.87
%		70.12	3.05	3.05	0	23.78	100	

The "Average" column is the "Total" divided by the "Number of households".

**Table 4:** Black bear attacks on humans over a 10-year timeframe (2009–2019).

Case	Sex	Village	Date	Victim People (Single/Group)	Place of Incident	Activity of Victim
1	Male	Kumdi	27 September 2014	Group	Forest	Timber harvesting
2	Male	Kumdi	18 September 2016	Group	Trail	Walking
3	Male	Kumdi	11 October 2019	Group	Trail	Walking
4	Female	Kumdi	11 October 2019	Single	Farmland	Crop harvesting
5	Male	Kumdi	11 October 2019	Group	Farmland	Chasing bear

## Data Availability

Dataset available on request from the authors.

## Appendix A

### Appendix A.1. Household Survey Questionnaire

- Name of the respondent:
- Address(village):
- Age:
- Sex:
- Residential status: ( ) Indigenous ( ) Migrant ( ) others
- Education level:
- Religion:

General description of respondent’s household:

Family size	Number: Male ( ) Female ( )
Primary source of income in priority order (a = most important, b = important, c = least important)	Agriculture: Business: Labor: Remittance: Service: Forest: Others:
Food sufficiency for	No of months:
Total land (Khet/bari)	…….. Ropani
Distance to the forest from the house (or time to reach forest)	(Estimation)…………….
Frequency of going to the forest	1 = frequently (several times in a month) 2 = occasionally (sometimes in a month) 3 = rarely (sometimes in a few months) 4 = never (no dependency on forest)
Purpose of going to the forest (a = most important, b = important, c = least important)	Fuel wood: Fodder: Timber: NTFP collections: others (specify):

### Appendix A.2. Problems Associated with Crop Damage

1. How much land do you cultivate, and which types of crops do you grow?

Own………..

Leased………

2. When do you cultivate your land? Which wild pest animals are responsible for crop damage? (Calendar of major crops)

S. N.	Crop	Sowing	Weeding	Harvesting Period	Crop damaging wild animals
1	Maize				
2	Wheat				
3	Barley				
4	Potato				
5	Buckwheat				
6	Bean				
7	Others				

3. Please fill in the following tables of agricultural production and loss by asking the respondents.

SN	Crop	Area of lands (Ropani)	Production (kg)		Loss of Crops (kg)		Local price of crop/kg	Crop damage by (Mention each responsive animal to crop loss and their depredation quantity) kg			
			2018	2019	2018	2019	2019	Black bear	Ghoral	Wild boar	Others
1	Maize										
2	Wheat										
3	Barley										
4	Potato										
5	Buckwheat										
6	Latte										
7	Bean										
8	Others										

4. Which way does damage occur? e.g., By eating [ ] by mauling [ ] both [ ]

5. In which time period does the black bear damage your crop? (Please tick one of the following) Day ( ) or Night ( )

1 = Early night (6 p.m.–9 .m.)
2 = Midnight (9 .m.–3 a.m.)
3 = Early morning (3 a.m.–6 a.m.)
4 = Day (6 a.m.–6 .m.)

6. Which month?

7. Which agricultural crop is preferred by black bears? (Please give code like mostly preferred 1 then 2 … 3)

a. Maize ( ) b. Wheat ( ) c. Potato ( ) d. Buckwheat ( ) e. other ( )

8. What are the preferred wild foods of black bears? (Please mention the local name of wild food).

### Appendix A.3. Problems Associated with Livestock Depredation

1. Major livestock holding and practices rearing.

S.N.	Livestock	Total no. of livestock	Number of livestock allowed to graze	Stall feeding no. of livestock	Grazing and stall feeding no.
1	Cow/Ox				
2	Buffalo				
3	Chauri/Yak				
4	Horse				
5	Sheep				
6	Goat				

2. For grazing, where do you take your livestock?

S.N.	Grazing Area	Seasons	
		Summer	Winter
1	Using/nearest forest		
2	Distance forest/grazing land		
3	Village area/agricultural field		

Using/nearest forest: that forest within reach of 1, or forests with a traditional right for use. Distance/grazing land: those forests or grazing land that need more than 1.5 h to reach. Village area/agricultural field: village periphery, village open land, public land, etc.

3. Grazing area and season

S.N.	Season	Grazing Places
1	Summer	
2	Winter	

4. Livestock depredation and loss

4.1 Livestock attacked for the last 5 years in your family/ household?

S.N.	Livestock	Total no. of attacks	Attacked by				Price of killed livestock
			Black Bear	Jackal	Wild boar	Others	
1	Cow/ Ox						
2	Buffalo						
3	Chauri/ Yak						
4	Horse						
5	Sheep						
6	Goat						

4.2 Time of spatial and temporal attack by black bear

S.N.	Livestock	Sex		Age	Attacked place (crop field, village, forest)	Attacked season (spring, summer, autumn, winter)	Attacked month	Attacked time (night, day)
		Male	Female					
1	Cow/Ox							
2	Buffalo							
3	Chauri/Yak							
4	Horse							
5	Sheep							
6	Goat							

### Appendix A.4. Problems Associated with Human Casualties

1. Have bears attacked you or a member of your family? Yes [ ] No [ ] If yes,

S.N.	Victim’s name	Sex	Age	Date of attack	Victim people (single/group)	Place of incident (forest, farmland, trail, others)	Activity of victim
							

2. Type of casualty: a. Minor injury b. Major injury c. Death

3. Do the victim/ family members get compensation for these types of casualties?

### Appendix A.5. Local Communities’ Attitudes toward Black Bear

1. Do you always want to see black bears in your surroundings/range lands? Do you feel good to see black bears?

Yes = 1 No = −1 Not sure = 0

2. Do you think the black bear should be conserved/ protected or have the right to live in the forest?

Yes = 1 No = −1 Not sure = 0

3. Do you think the black bear improves the environment’s balance/stability/quality?

Yes = 1 No = −1 Not sure = 0

4. Would you support conservation authorities with time or money if they started to conserve black bears in your area?

Yes = 1 No = −1 Not sure = 0

5. Should we teach our children to help protect black bears?

Yes = 1 No = −1 Not sure = 0
